# Consensus on the Use of Mefenamic Acid in Pediatric Practice (MAPP): Perspectives From Indian Pediatricians

**DOI:** 10.7759/cureus.88412

**Published:** 2025-07-21

**Authors:** Uday Pai, Ravishankar A Venkata, Abhay Shah, Alok Gupta, Antony Clement, Arun Wadhwa, Ashwani Kumar, A Somasundaram, Girish Charde, K K Joshi, Mukesh Sanklecha, Mylapore V Suresh Kumar, Praveen Gokhale, Rajeev Agarwal, Raju Shah, Ritesh Chhabra, Sanjay Niranjan, Shilpa Dudhgaonkar, Srikrishna Surampudi, Sumitha Nayak, Vibhor V Borkar

**Affiliations:** 1 Department of Pediatrics, Sai Kutir Clinic, Mumbai, IND; 2 Department of Pediatrics, Dr. Ravishankar Clinic, Chennai, IND; 3 Department of Pediatrics, Children's Hospital and Pediatric Infectious Diseases Centre, Ahmedabad, IND; 4 Department of Pediatrics, Pediatric Specialties Clinic, Jaipur, IND; 5 Department of Pediatrics, Indira Gandhi Co-Operative Hospital, Kochi, IND; 6 Department of Pediatrics and Child Health, Wadhwa Clinic, Delhi, IND; 7 Department of Pediatric Medicine, Adesh Medical College and Hospital, Ambala, IND; 8 Department of Pediatrics, D'Soul Child Development Center, Chennai, IND; 9 Department of Pediatrics, New Health City Hospital and Research Center, Nagpur, IND; 10 Department of Pediatrics, Prasanthi Hospital, Manjeri, IND; 11 Department of Pediatrics, Bombay Hospital Institute of Medical Sciences, Mumbai, IND; 12 Department of Pediatrics, Maternity, Child, and Family Health Clinic, Chennai, IND; 13 Department of Pediatrics, Jupiter Hospital, Thane, IND; 14 Department of Pediatrics, Shree KKasturi Medicare Pvt. Ltd., Mira Bhayandar, IND; 15 Department of Pediatrics, Ankur Institute of Child Health, Ahmedabad, IND; 16 Department of Pediatrics, Chhabra Hospital, Ludhiana, IND; 17 Department of Pediatrics, Neo Child Care, Lucknow, IND; 18 Department of Pediatrics, Srinivas Children's Hospital, Pune, IND; 19 Department of Pediatrics, Apollo Medical College, Hyderabad, IND; 20 Department of Pediatrics, The Children's Clinic, Bengaluru, IND; 21 Department of Pediatric Hepatology, Gastroenterology, and Transplant Medicine, Gleneagles Hospital, Mumbai, IND

**Keywords:** antipyretic, febrile seizures, mefenamic acid, nlrp3 inhibition, nsaids, pediatrics

## Abstract

Fever and pain are among the most common reasons for pediatric consultations, requiring effective and safe management strategies. Mefenamic acid, a well-established member of the nonsteroidal anti-inflammatory drug (NSAID) class, offers distinct advantages due to its unique pharmacological profile, including preferential cyclooxygenase-2 (COX-2) inhibition, E-type prostanoid (EP) receptor blockade, and nucleotide-binding domain, leucine-rich-containing family, pyrin domain-containing-3 (NLRP3) inflammasome inhibition. These properties enable it to address both inflammatory and non-inflammatory fevers, providing comprehensive symptom relief. Despite its proven efficacy, the absence of pediatric-specific guidelines and perceived concerns about safety have limited its routine use in pediatric practice.

This consensus paper, developed through a structured modified Delphi process involving 21 expert pediatricians across India, aims to address these gaps. The paper evaluates the safety, efficacy, and clinical applications of mefenamic acid, providing evidence-based best-practice recommendations. Highlights include its superior antipyretic efficacy compared to paracetamol and ibuprofen, with preclinical and limited clinical data suggesting potential antiviral activity, and a possible role in the management of febrile seizures and inflammatory conditions. While mefenamic acid demonstrates a favorable safety profile with appropriate use, further research is necessary to strengthen the evidence on long-term safety and expand its therapeutic scope. This consensus provides a comprehensive framework for optimizing the use of mefenamic acid in pediatric practice, ensuring improved patient outcomes. The long-term safety of mefenamic acid in children is still unclear, due to the limited availability of robust, longitudinal safety data. Future research should prioritize well-powered clinical trials, particularly in children aged six months to five years, using standardized outcome measures and long-term follow-up protocols. Addressing these evidence gaps is important to inform safe and effective use in pediatric practice.

## Introduction and background

Background and clinical relevance of mefenamic acid

Fever and pain are among the most frequently encountered symptoms in pediatric practice, making them significant contributors to the healthcare burden in children. Fever, in particular, remains one of the primary reasons for pediatric consultations [1,2]. Annually, approximately 70% of preschool-aged children experience episodes of fever, with about 40% of these cases seeking medical care [3]. About 15-25% of children with fever need emergency visits to the clinic [1].

Nonsteroidal anti-inflammatory drugs (NSAIDs) are the cornerstone of managing fever and pain in children, with paracetamol, ibuprofen, and mefenamic acid being the choices of drugs [2]. Although paracetamol and ibuprofen have traditionally been the most widely used over-the-counter antipyretics, mefenamic acid has been used frequently due to its distinguished unique pharmacological profile, which includes preferential cyclooxygenase-2 (COX-2) inhibition, E-type prostanoid (EP) receptor blockade, and nucleotide-binding domain, leucine-rich-containing family, pyrin domain-containing-3 (NLRP3) inflammasome inhibition [2,4]. These unique mechanisms of action may offer advantages over paracetamol and ibuprofen for managing fever and pain in children, particularly in cases where these antipyretics may show suboptimal efficacy or safety [2].

Mefenamic acid is an effective option for antipyresis and inflammation, with evidence supporting its role in pediatric febrile illnesses [2]. Studies have highlighted its clinical uses, efficacy, and safety profile, along with its potential to address inflammatory fever and febrile seizures through its NLRP3 inhibitory action [5]. Additionally, emerging evidence suggests potential antiviral properties, making mefenamic acid a valuable option during viral infections [6]. Its usefulness during the COVID-19 pandemic and other emerging evidence have emphasized its therapeutic potential beyond fever and pain management [7]. Also, despite its routine use in clinical practice, there is a lack of formal recommendations for prescribing mefenamic acid in children, and concerns persist regarding its safety, particularly related to potential gastrointestinal (GI) irritation, renal adverse effects, and the risk of seizures reported in isolated cases. These concerns, often based on limited or outdated evidence, contribute to variability in prescribing practices.

Unmet need

Despite established efficacy as an antipyretic, analgesic, and anti-inflammatory NSAID, the use of mefenamic acid in pediatrics remains limited by significant gaps in knowledge and clinical practice. A paucity of pediatric-specific clinical guidelines, such as those from the Indian Academy of Pediatrics (IAP), and robust evidence from large-scale studies in this patient population hinder consistent and evidence-based use of mefenamic acid in pediatric practices, leading to considerable variability in dosing, indications, and treatment durations. Misconceptions around its safety further discourage its routine clinical use. Additionally, the potential use of mefenamic acid in conditions beyond acute pain and fever remains underexplored. These challenges are further compounded by limited awareness among healthcare providers, which collectively contributes to the underutilization of mefenamic acid in pediatric care. Additionally, there is a lack of guidelines that align with emerging evidence and balance clinical benefits against the associated risk profile.

Objectives

This consensus paper seeks to address the challenges of the use of mefenamic acid in pediatric practice by aligning expert opinions and current evidence through a comprehensive review of the literature and the development of evidence-based best-practice statements. The key objectives include evaluating the safety, efficacy, and clinical applications of mefenamic acid, bridging gaps between clinical evidence and practice, and proposing future research directions to expand its therapeutic potential.

Scope and target audience

The scope of the consensus paper is to provide best-practice recommendations for the use of mefenamic acid in pediatric practices. These recommendations are intended for pediatricians, general practitioners, pharmacologists, and other healthcare providers involved in pediatric care.

Approach to developing the consensus paper

Decisions on best practices in medical education should be evidence-based, but such evidence is often lacking. Consequently, reliance on expert judgment, if informal, may lack credibility. Formal consensus methods provide a structured approach to synthesizing expert opinions, enabling measurement of agreement or resolution of disagreements [8]. For this purpose, we employed a three-step modified Delphi method as a reliable approach for addressing defined clinical problems by considering insights from an expert panel [9]. The consensus was developed during an advisory board meeting held on November 24, 2024, in Mumbai, India.

Assembly of the Expert Panel

Experts representing general pediatricians and neonatologists were invited to participate in the consensus development. A total of 21 experts from Pan-India were finally included in the expert panel. A senior pediatrician chaired the panel and monitored and drove the consensus development process. The remaining 20 experts were divided into four groups, with five experts in each group.

All panel members contributed equally to the discussions and actively participated in every phase of the consensus development process. Each expert maintained independence in decision-making, ensuring that their judgments were unbiased and free from any conflicts of interest.

Literature Review and Evidence Compilation Strategy and Criteria

Four groups of experts were assigned one topic each for a literature review on mefenamic acid. Group I examined the historical and pharmacological versatility of mefenamic acid, while Group II explored the clinical evidence supporting its use in pediatric practice. Group III assessed its benefit-risk profile, and Group IV delved into recent advances and future potential. The groups were requested to conduct a thorough literature search, form the initial consensus statements, and augment and consolidate the relevant evidence supporting these statements on the assigned topics.

Each group conducted an extensive review of peer-reviewed articles, clinical trials, and pharmacological studies relevant to their topic. The literature search was conducted using MEDLINE, Embase, and Google Scholar databases (see Appendices). Articles were limited to those published in English between January 2011 and November 2024. Search terms were predefined to ensure consistency and relevance. A backward citation search was also performed on selected articles to identify additional relevant publications and the literature supporting important information published prior to the specified time frame mentioned above.

No formal quantitative synthesis (meta-analysis) was performed; the evidence was compiled narratively to support the development of consensus statements. 

Delphi Polling Process and Criteria for Consensus

During the physical meeting, all four working groups presented consolidated scientific literature on the topic assigned to them. Following the presentations and discussions, Delphi polling was done. Statements finalized by each group were considered for the polling process. A total of 22 statements were included in the first round of Delphi polling. Consensus was obtained through iterative polling using a five-point Likert scale (strongly agree, agree, neither agree nor disagree, disagree, or strongly disagree) using the electronic voting platform (polleverywhere.com). Statements were considered to reach a consensus if 75% or more of the panel members agreed or strongly agreed with it and were finalized. The Delphi polling was conducted by the chairperson, who did not participate in the voting to maintain neutrality. The remaining 20 expert panel members independently rated each consensus statement using a five-point Likert scale. During the first round, 11 statements achieved the threshold of 75% and were considered final without further deliberations. The rejected 11 statements were considered for the second round of the Delphi polling for further deliberations. During the second round, disagreements on the rejected statement were discussed and resolved by rewording or rephrasing the statements with the incorporation of suggestions from the experts. The polling was carried out for these statements, where three statements reached a consensus and were considered final without further deliberations. In the second round, eight statements were rejected and considered for the final round (third round) of polling with further deliberations. During the third round, the eight rejected statements were discussed for disagreements and were reworded and rephrased for polling; however, all eight statements were rejected by the experts, with less than 75% of panelists voting in favor of these statements. At the end of the Delphi polling process, a total of 14 statements were accepted as best-practice statements.

**Figure 1:**
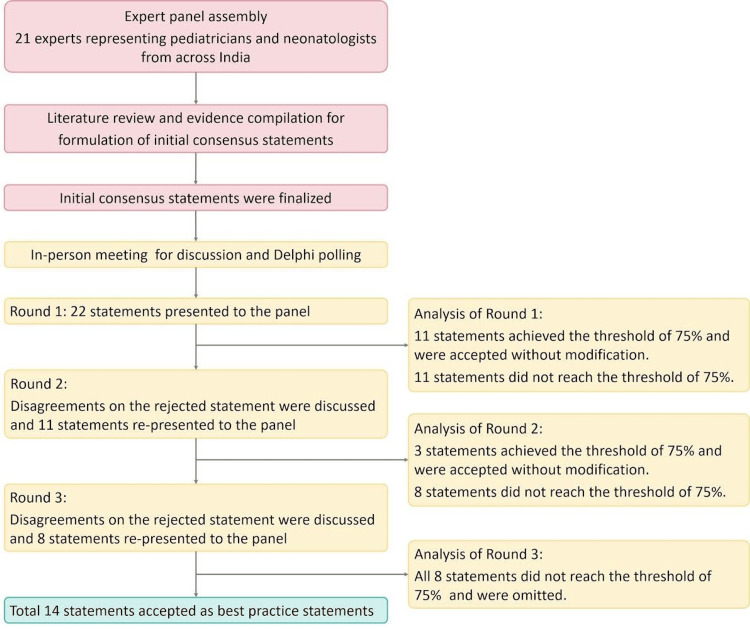
Flow diagram of the consensus development process and steps in the Delphi polling process

Figure 1 summarizes the process of developing the consensus paper and Delphi polling.

## Review

Historical and pharmacological insights

History of Mefenamic Acid in Clinical Practice

Mefenamic acid, a derivative of N-phenylanthranilic acid and a member of the "fenamate" family of NSAIDs, was first introduced as a pharmaceutical agent in 1962. Developed during the 1950s through extensive research on fenamates, mefenamic acid emerged as a key representative of this drug class, demonstrating superior efficacy and safety in clinical trials [10]. It quickly gained popularity, becoming one of the most widely prescribed NSAIDs by the 1980s, and is in clinical use globally, including in Europe, Southeast Asia, and India, with its use continuing to expand due to its versatility in treating various conditions [4,10].

Mechanism of Action and Unique Properties of Mefenamic Acid

Mefenamic acid is an NSAID that exerts its therapeutic effects through the preferential inhibition of cyclooxygenase (COX) enzymes, centrally and peripherally, enabling comprehensive fever, inflammation, and pain management [11-13]. Mefenamic acid is considered a competitive, time-dependent, and reversible inhibitor of both COX-1 and COX-2 enzymes [10], with a 12.5-fold preferential selectivity for COX-2 [14]. This preferential nature of mefenamic acid enables potent pharmacological activity while minimizing GI side effects commonly associated with non-selective COX inhibition [2,4].

Mefenamic acid blocks all major fever-producing pathways, including prostaglandins and pyrogenic cytokines [2,4]. Mefenamic acid effectively blocks fever by inhibiting both neuronal and humoral pathways. The neuronal pathway for fever involves direct neural signals, primarily transmitted through the vagus nerve, from immune cells to the brain [15,16]. Mefenamic acid blocks this pathway by reducing the peripheral activity of prostaglandin E2 (PGE2) [17,18]. In the humoral pathway of fever, circulating pro-inflammatory and pyrogenic cytokines, such as interleukin-1 beta (IL-1β), IL-6, and tumor necrosis factor alpha (TNF-α), cross the blood-brain barrier and stimulate the production of PGE2 in the preoptic area of the hypothalamus, elevating the set point temperature of the body [16]. Mefenamic acid also blocks the humoral pathway by inhibiting the production of PGE2 centrally and reducing the levels of circulating cytokines [2,4].

What sets mefenamic acid apart from other NSAIDs is its ability to block EP receptors, which inhibits pre-formed prostaglandins [4,19]. The dual action, targeting both newly synthesized and pre-existing prostaglandins, ensures a potent and robust antipyretic, anti-inflammatory, and analgesic response.

Beyond its COX-mediated effects, mefenamic acid exhibits novel anti-inflammatory actions through the inhibition of the NLRP3 inflammasome, a key player in various inflammatory and autoimmune conditions [4,20]. By selectively blocking the volume-regulated anion channels (VRAC) and volume-modulated transient receptor potential melastatin 2 (TRPM2) channels, mefenamic acid prevents IL-1β release [21,22]. These effects are independent of its COX enzyme inhibition, highlighting its multifaceted mechanism of action.

The combination of COX enzyme inhibition, EP receptor blockade, and NLRP3 inflammasome inhibition positions mefenamic acid as a unique therapeutic option in the NSAID category. Its ability to synergize with inhibitors of various inflammatory pathways provides a superior level of control over inflammation, pain, and fever compared to single-mechanism agents (Table 1) [4,19-22]. Additionally, emerging evidence of its antiviral potential further underscores its versatility in managing complex conditions involving fever, inflammation, and pain.

**Table 1:** Unique mechanisms of action of mefenamic acid and their benefits COX: cyclooxygenase; EP: E-type prostanoid; NLRP3: nucleotide-binding domain, leucine-rich-containing family, pyrin domain-containing-3

Unique properties	Benefits
Preferential COX-2 inhibition	Potent pharmacological activity and reduced gastrointestinal side effects over non-selective COX inhibitors
Blocks EP receptors	Inhibition of pre-formed prostaglandins
Inhibits NLRP3 inflammasome	Minimizes the formation of pyrogenic and pro‑inflammatory cytokine levels
Acts on both neuronal and humoral fever pathways	Potent antipyretic activity

Mechanistic Difference of Mefenamic Acid Compared to Paracetamol and Ibuprofen

Mefenamic acid and paracetamol differ significantly in their mechanisms of action. Paracetamol acts primarily through central mechanisms, inhibiting prostaglandin synthesis in the brain, but it lacks peripheral action and exhibits no anti-inflammatory properties [2,23]. This limited scope can result in suboptimal control of fever, particularly in conditions with an inflammatory component [2]. In contrast, mefenamic acid is an NSAID demonstrating dual action, targeting both central and peripheral pathways. Also, it blocks EP receptors, rendering pre-existing prostaglandins inactive. This is more effective in fever reduction but also offers substantial anti-inflammatory benefits over paracetamol [2,23]. While paracetamol is widely used for its ability to reduce fever, its shorter antipyretic effect, coupled with its lack of anti-inflammatory activity, highlights the advantages of mefenamic acid in managing conditions where inflammation exacerbates disease severity.

On the other hand, while both mefenamic acid and ibuprofen are NSAIDs, ibuprofen is a non-selective COX inhibitor, whereas mefenamic acid preferentially inhibits COX-2 [2,4,24]. Being a non-selective COX inhibitor, ibuprofen is associated with a higher risk of GI side effects [24]. Unlike mefenamic acid, ibuprofen lacks EP receptors and the NLRP3 inflammasome inhibitory activity [2,4].

Comparative Pharmacokinetics of Mefenamic Acid, Paracetamol, and Ibuprofen

The comparative pharmacokinetics of mefenamic acid, paracetamol, and ibuprofen revealed distinct characteristics that influence their efficacy, safety, and clinical applications. A summary of these pharmacokinetic parameters in adults is presented in Table 2 [25-32].

**Table 2:** Comparative PK with mefenamic acid, paracetamol, and ibuprofen BA: bioavailability; PB: protein binding; PK: pharmacokinetic; T_max_: time to peak plasma concentration; V_d_: volume of distribution

PK property	Mefenamic acid	Paracetamol	Ibuprofen
Absorption	BA: ~90%; T_max_: 2-4 h	BA: ~80%; T_max_: ~2 h	BA: 80%; T_max_: 1-2 h
Distribution	V_d_: 1.06 L/kg; PB: >90%	V_d_: 0.9 L/kg; PB: low	V_d_: 0.12-2 L/kg; PB: 90-99%
Metabolism	Hepatic; mainly via CYP2C9	Hepatic; mainly via CYP2C9 and minor via CYP2E1	Hepatic; rapidly metabolized via CYP2C9
Excretion	Renal: 52%; fecal: 20%	Renal: 90%	Renal: 45-79%
Half-life (t_1/2_)	2-4 h	1-4 h	1.8-2.44 h

Mefenamic acid is well absorbed orally, reaching peak plasma concentrations (Cmax) in 2-4 hours (tmax), with a half-life (t1/2) of 2-4 hours. It binds over 90% to plasma proteins and is primarily metabolized by CYP2C9, with 50% excreted in urine and 20% in feces [25,26]. Paracetamol, with ~80% bioavailability, peaks at two hours (Tmax), has low protein binding, and has a half-life of 1-4 hours. It is mostly metabolized in the liver through glucuronidation and sulfation, with 90% excreted in urine [27-29]. CYP-mediated metabolism of paracetamol results in the production of the reactive metabolite N-acetyl-p-benzoquinoneimine (NAPQI/NABQI), which is a crucial determinant of its associated hepatotoxicity [30]. Ibuprofen is quickly absorbed, reaching Cmax in 1-2 hours, with a half-life of 1.8-2.44 hours, and binds extensively to plasma proteins. It undergoes liver metabolism, with 45-79% excreted in urine within 24 hours [26,31,32]. Differences in absorption, half-life, and metabolism among these drugs influence their onset and duration of action and suitability for specific patient needs.

While these pharmacokinetic data are derived from adult studies, it is important to note that pediatric pharmacokinetics can differ significantly due to age-related variations in drug absorption, distribution, metabolism, and excretion. Factors such as immature liver enzyme systems, altered plasma protein binding, and differences in renal clearance may affect drug exposure and response in children. However, comprehensive pediatric-specific pharmacokinetic data for mefenamic acid, and to a lesser extent for paracetamol and ibuprofen, remain limited. This knowledge gap highlights the need for targeted pharmacokinetic studies in pediatric populations to inform age-appropriate and evidence-based dosing strategies.

Dosage Guidelines for Pediatric Patients

The recommended dosage of mefenamic acid in pediatric patients is 4-6.5 mg/kg body weight every eight hours, not exceeding three doses per day [33]. Clinical studies have demonstrated the safe use of mefenamic acid in children over six months of age at dosages ranging from 4 to 6.5 mg/kg per dose [2,23,33]. In children under 12 years of age, mefenamic acid is typically administered in the form of suspension formulations [34], while children aged 12-18 years may use 250 mg dispersible tablets or 500 mg tablets [35]. Mefenamic acid should be taken with food and adequate water to minimize GI irritation and optimize absorption [36].

Indications

Mefenamic acid is approved for various indications in pediatric patients, offering antipyretic, analgesic, and anti-inflammatory benefits. It is commonly used as an antipyretic for managing fever in children above six months of age and for addressing pyrexia associated with inflammatory pain [2]. Additionally, mefenamic acid serves as an effective anti-inflammatory analgesic, providing symptomatic relief for conditions such as rheumatoid arthritis, osteoarthritis, headaches, muscular pain, dental pain, post-traumatic pain, and post-operative pain [37]. In older children and adolescents, it is also indicated for gynecological conditions like primary dysmenorrhea [37]. Experts also suggested the indication of mefenamic acid for pain associated with otitis media and sinusitis, as well as for pain associated with smooth muscle spasms like renal colic, intestinal, and biliary colic.

While prescribing mefenamic acid in children for febrile illness, the focus should be on reducing discomfort rather than the temperature [38]. Both national and international guidelines recommend that the management of fever with antipyretics should focus on improving the child's overall condition and should be administered only when the child shows discomfort, regardless of a specific body temperature threshold [39]. Discomfort in febrile illness is mainly due to associated symptoms such as myalgia, sore throat, or headache. Mefenamic acid, with its dual antipyretic and analgesic properties, helps to enhance comfort by relieving pain, reducing irritability, and promoting better feeding and hydration [33]. This holistic approach not only mitigates discomfort but also reduces the risk of dehydration associated with febrile conditions. The long-lasting antipyretic action of mefenamic acid provides sustained relief, aligning with the goal of improving comfort in febrile children [2].

Clinical efficacy of mefenamic acid in pediatric patients

Mefenamic acid has emerged as a valuable therapeutic option in managing febrile illnesses and pain in pediatric patients. With its established antipyretic, analgesic, and anti-inflammatory properties, mefenamic acid provides rapid and sustained symptom relief, addressing key challenges in fever management. Although fewer studies have investigated the antipyretic efficacy of mefenamic acid in pediatric patients compared to adults, existing literature highlights its superior effectiveness and better tolerability relative to paracetamol and ibuprofen in reducing fever, as summarized in Table 3 [23,40-43]. Across various clinical trials, mefenamic acid has consistently shown a faster onset of action, greater temperature reduction, and sustained antipyretic effects (Table 3).

**Table 3:** Studies evaluating the antipyretic efficacy of mefenamic acid in pediatric patients

Study	Design	Participants	Interventions	Key findings
Khubchandani et al. (1995) [41]	Randomized, open-label, comparative study	87 children with fever (100-105°F)	Paracetamol 10 mg/kg (n=29), ibuprofen 7 mg/kg (n=29), mefenamic acid 6.5 mg/kg (n=29)	Mefenamic acid achieved a higher temperature reduction compared to paracetamol and ibuprofen. Significant antipyretic effect observed over 2-4 hours (p<0.05).
Kunkulol et al. (2013) [23]	Prospective, active treatment-controlled study	124 pediatric patients with fever (>38.5°C)	Paracetamol 15 mg/kg (n=62), mefenamic acid 4 mg/kg (n=62)	Mefenamic acid demonstrated higher efficacy and faster fever reduction at 1, 4, and 6 hours compared to paracetamol (p<0.05). Suggested as a superior alternative with equal tolerability.
Reddy et al. (2020) [40]	Randomized, open-label study	180 children (1 month-14 years) with fever of 100-105°F	Paracetamol 15 mg/kg (n=60), ibuprofen 10 mg/kg (n=60), mefenamic acid 8 mg/kg (n=60)	Mefenamic acid showed faster onset and greater temperature reduction at 2 hours and 6 hours compared to paracetamol and ibuprofen. Sustained effect observed at 24 hours.
Dabholkar (2002) [43]	Open-label, prospective study	160 children (≥2 months old) with fever 100°F and above	Single dose of mefenamic acid 6.5 mg/kg	Rapid onset with temperature normalization by 3 hours; sustained reduction for 8 hours. Well tolerated, with 6% of children experiencing mild gastrointestinal side effects like nausea, vomiting, and abdominal discomfort.
Loya et al. (2022) [42]	Randomized controlled trial	165 febrile children (6 months-5 years) with an axillary temperature of 100.4°F or above	Standard-dose paracetamol (15 mg/kg), high-dose paracetamol (20 mg/kg), mefenamic acid (6 mg/kg)	Standard-dose paracetamol had a slower and shorter effect compared to mefenamic acid. Mefenamic acid was shown to have superior antipyretic activity.

In an open-label study by Dabholkar, 160 children aged two months and older with fever ≥100°F were evaluated [43]. A single dose of mefenamic acid (6.5 mg/kg) produced an antipyretic effect within one hour. Temperatures normalized by the third hour, and the fever reduction was sustained for up to eight hours [43]. This prolonged efficacy is also supported by a study from Reddy et al. involving 180 children aged one month to 14 years [40]. The authors observed that mefenamic acid provided the fastest onset of action, with a statistically significant temperature reduction compared to paracetamol and ibuprofen occurring as early as two hours. At six hours also, the fall in temperature with mefenamic acid was maximum and statistically significant compared to paracetamol and ibuprofen. Furthermore, it also demonstrated a sustained reduction in the baseline mean temperature at the end of 24 hours, compared to paracetamol and ibuprofen [40]. In a comparative study by Khubchandani et al., mefenamic acid (6.5 mg/kg) achieved a temperature drop at four hours, significantly greater than paracetamol and ibuprofen [41]. In this study, mefenamic acid exhibited significantly superior antipyretic activity compared to paracetamol (p<0.05) over four hours and ibuprofen (p<0.05) within the 2-4-hour range [41]. Reddy et al. have also reported a maximal temperature reduction of mefenamic acid [40]. A study by Kunkulol et al., involving 124 children, found that mefenamic acid (4 mg/kg) provided significantly greater (p<0.05) antipyretic activity at one, four, and six hours compared to paracetamol (15 mg/kg). Moreover, the study concluded that mefenamic acid enabled faster recovery from fever, reinforcing its role as a more potent and effective antipyretic option [23]. Advantages of mefenamic acid are further highlighted in a randomized controlled trial by Loya et al. The trial found that standard-dose paracetamol (15 mg/kg) has a slower onset and shorter antipyretic effect compared to mefenamic acid [42]. The sustained antipyretic action of mefenamic acid also significantly reduces the dosing frequency in fever management, thereby improving therapeutic adherence. The sustained action not only minimizes the disruption of sleep for pediatric patients and their caregivers but also addresses concerns associated with "fever phobia" among parents. By ensuring sustained fever control, mefenamic acid allows for uninterrupted sleep, promoting comfort and psychological reassurance in managing febrile conditions [2].

The analgesic and anti-inflammatory activity of mefenamic acid is well documented in the adult population. Although mefenamic acid is used in children for the management of pain and inflammatory diseases, there is a lack of literature describing its analgesic or anti-inflammatory efficacy in pediatric patients. The Mefenamic Acid versus Paracetamol for pain prophylaxis during vaccination (MAP VaC) study, a triple-blind, randomized controlled trial involving 135 children, evaluated mefenamic acid (4 mg/kg) as pre-vaccination pain prophylaxis [44]. Significant reductions in Face, Legs, Activity, Cry, and Consolability (FLACC) pain scores were found at administration (p=0.010) and 15 minutes post-vaccination (p=0.014) compared to placebo [44]. However, no differences were observed in fever incidence, severity, or antipyretic use within 24 hours. Despite promising results for alleviating needle pain, the prophylactic use of antipyretics is controversial due to concerns about reduced vaccine responses, and expert guidelines recommend reserving such treatments for post-vaccination complications [33,45].

Overall, current clinical evidence supports the superior antipyretic efficacy of mefenamic acid in pediatric patients, offering rapid and sustained fever control with comparable tolerability to other commonly used agents, including paracetamol and ibuprofen, making it a valuable option in managing pediatric febrile conditions. However, despite these promising findings, gaps in current evidence remain, particularly regarding its analgesic and anti-inflammatory efficacy in pediatric populations. Also, limited data are available for its role in specific conditions beyond fever management. Future research should focus on addressing these gaps through large-scale, well-designed studies to further define the therapeutic scope of mefenamic acid in pediatric practice.

Safety profile of mefenamic acid

Over the past six decades, mefenamic acid has demonstrated a well-established safety profile in clinical trials, including pediatric studies and even among preterm infants. Renowned for its favorable tolerability compared to ibuprofen and paracetamol, mefenamic acid is considered one of the safest NSAIDs [46].

Comparative Safety of Mefenamic Acid

In clinical studies in pediatric patients, mefenamic acid has shown a favorable safety and tolerability profile. Dabholkar reported that 94% of children tolerated mefenamic acid well, with no adverse effects observed [43]. Across studies, its tolerability was found to be comparable to that of paracetamol and ibuprofen, further supporting its safety for pediatric use [23,40-42].

Better GI Safety

Class action of NSAIDs by COX inhibition can also lead to GI irritation and ulcer formation. This risk arises from the inhibition of COX-1 in the gastric mucosa, which impairs protective prostaglandin synthesis [14]. The inhibition of gastric PGE2 synthesis by NSAIDs has also been found to strongly correlate with COX-1 inhibitory potency in the blood (p<0.001) and COX-1 selectivity (p<0.01) but not with COX-2 inhibitory potency [14]. In this context, COX-2 selective or preferential NSAIDs may improve effectiveness while minimizing significant GI side effects [14].

Due to its preferential COX-2 inhibitory properties, mefenamic acid is associated with only minor GI symptoms when used appropriately. Studies have shown that administering the drug with food and ensuring adequate hydration significantly reduces the risk of GI side effects associated with NSAIDs [36]. GI symptoms may resolve in most cases when mefenamic acid is taken with meals and a sufficient volume of water without affecting the bioavailability of the drug [47]. Studies have also demonstrated the comparative GI safety advantage of mefenamic acid with lower rates of GI-related hospitalizations [48].

Lower Risk of Nephrotoxicity and Hepatotoxicity

Nephrotoxicity is a recognized class effect of NSAIDs, arising from their core mechanism of COX enzyme inhibition. By interfering with the conversion of arachidonic acid into vasodilatory prostaglandins such as PGE2 and prostaglandin I2, also known as prostacyclins (PGI2), NSAIDs disrupt renal autoregulation, reducing renal perfusion [49]. The renal toxicity is particularly observed in high-risk scenarios involving chronic NSAID use. Moreover, the nephrotoxic effects of NSAIDs are typically transient and reversible upon discontinuation [49].

Factors such as advanced age, comorbidities, and concurrent use of renin-angiotensin blockers or diuretics heighten susceptibility to NSAID-induced nephrotoxicity. Furthermore, conditions like salt restriction, volume depletion, and renovascular hypertension, which induce a high-renin state and elevate macula densa COX-2 expression, further amplify this risk [49,50].

There is no direct evidence linking mefenamic acid to hepatotoxicity. Hepatic dysfunction is considered a rare adverse event associated with the use of mefenamic acid, further supporting its overall safety profile in pediatric patients when used appropriately [10].

Central Nervous System (CNS) Safety and Neuroprotective Effects

CNS toxicity associated with mefenamic acid has been reported primarily in cases of overdose, with no significant concerns observed at therapeutic doses. Evidence indicates a dose-dependent risk of convulsions when mefenamic acid is consumed in higher doses [51]. Overdoses as low as 2.5 g have been linked to convulsions, while moderate to severe CNS symptoms were observed at doses of 3.5 g or higher, both significantly exceeding the recommended therapeutic range [51]. At higher doses, mefenamic acid can lower the seizure threshold, particularly in individuals with pre-existing seizure disorders, posing a risk of seizures after overdose [51].

Interestingly, at therapeutic doses, mefenamic acid exhibits neuroprotective properties [52]. Research has highlighted its anticonvulsant potential in conditions such as febrile seizures and febrile infection-related epilepsy syndrome (FIRES) [52]. Fenamate NSAIDs, including mefenamic acid, have shown antiseizure actions that are independent of their well-established anti-inflammatory effects [53]. It has been demonstrated to modulate GABA "A" (gamma-aminobutyric acid) receptors, with both potentiating and inhibitory actions observed in vitro [54]. Mefenamic acid may reduce excitotoxicity by limiting glutamate release, thereby contributing to its neuroprotective effects [55]. Studies also showed NLRP3 inflammasome inhibition as a possible mechanism for the neuroprotective action of mefenamic acid, considering the key involvement of NLRP3 inflammasome in the development of neuroinflammation [56]. Emerging evidence suggests that mefenamic acid may exert neuroprotective actions beyond these mechanisms, warranting further research into its potential role in managing neurological conditions [52].

Safety During Pregnancy and Lactation

Mefenamic acid is a Category C drug, with animal studies showing adverse effects on the fetus and no well-controlled studies in pregnant women. It should be used only if the benefits outweigh the risks, avoiding use in the third trimester and consulting a healthcare provider before use [57].

The American Academy of Pediatrics (AAP) considers mefenamic acid safe for breastfeeding, with no reported adverse effects on nursing infants or lactation [58]. Though earlier reports regarded mefenamic acid as safe for use during lactation [59], further studies are needed to assess its long-term safety, especially in preterm infants [60].

Effect of Mefenamic Acid on Platelet Activity

The relationship between NSAIDs and bleeding risk is well documented, with most non-selective NSAIDs increasing the risk of bleeding by fourfold [61]. This is primarily attributed to the inhibition of COX-1, which reduces thromboxane A2 (TXA2) production, a critical factor for platelet aggregation and clot formation. Non-selective NSAIDs, such as ibuprofen, inhibit both COX-1 and COX-2 enzymes, leading to weakened platelet function and a heightened risk of bleeding, including GI bleeding [62,63]. Contrary to the general profile of NSAIDs, mefenamic acid has demonstrated a unique and favorable impact on platelet function. As a preferential COX-2 inhibitor, mefenamic acid spares TXA2 production, thereby preserving platelet function and avoiding the antiplatelet effects commonly seen with non-selective COX inhibitors [64]. Additionally, it has shown the ability to improve platelet aggregation and degranulation while promoting increased vasoconstriction, contributing to its hemostatic benefits [65]. Notably, mefenamic acid has been associated with a significant reduction of up to 40% in menorrhagia-related bleeding, highlighting its potential role in managing bleeding disorders without increasing overall bleeding risk [65]. Overall, unlike other NSAIDs, mefenamic acid is a safer choice regarding platelet function. Its ability to reduce bleeding in conditions like menorrhagia further distinguishes it from other NSAIDs.

Long-Term Safety of Mefenamic Acid in Children

The long-term safety of mefenamic acid in children remains uncertain due to a lack of robust studies or data in pediatric populations. While its long-term safety has been documented in adults [66], the absence of comprehensive research, particularly involving larger pediatric cohorts, leaves this aspect unexplored. Further studies are needed to evaluate its safety profile over extended use in children to ensure informed clinical decision-making.

Overall, mefenamic acid offers a comprehensive safety profile, distinguishing itself as a reliable and well-tolerated NSAID for pediatric use. Its favorable GI, renal, and hepatic safety, combined with its unique neuroprotective and platelet-sparing properties, underscores its therapeutic versatility. However, the absence of long-term safety data in children and the need for careful avoidance of concomitant NSAID use highlight areas requiring further investigation. Continued research into its long-term effects and pharmacological nuances in pediatric populations will help optimize its clinical utility while ensuring the highest standards of patient safety.

Emerging evidence and future potential

Role of NLRP3 Inflammasome as a Therapeutic Target

NLRP3 inflammasome plays a crucial role in the innate immune response, and activation of the NLRP3 inflammasome occurs in response to infection or cellular injury, wherein the inflammasome components oligomerize and assemble, leading to the activation of procaspase-1 into its active form. This activated form of caspase-1 facilitates the conversion of pro-inflammatory cytokines IL-1β and IL-18 into their active forms, driving a potent inflammatory response [22,67,68]. These inflammasomes are also essential in modulating immune responses across a wide range of physiological and pathological conditions, including metabolic disorders, tumorigenesis, and autoimmune diseases [69]. While these mechanisms are important for host defense against pathogens and managing cellular stress, aberrant activation can result in excessive inflammation and tissue damage [22]. Dysregulated or excessive activation of the NLRP3 inflammasome is implicated in various acute and chronic inflammatory, immune, and metabolic disorders, making it a promising therapeutic target [69].

In pediatric conditions such as febrile seizures, NLRP3 inflammasome activity is increasingly recognized as a critical factor. Elevated levels of NLRP3 and IL-1β in children with febrile seizures suggest a direct role of the NLRP3/IL-1β axis in the pathogenesis of these seizures [2,70]. Liu et al. reported significantly elevated NLRP3 and IL-1β levels in children with febrile seizures compared to fever-only controls (both p<0.05), regardless of seizure duration. A strong correlation between IL-1β and NLRP3 levels (r=0.787; p<0.001) highlights the NLRP3/caspase-1/IL-1β pathway's role in febrile seizure pathogenesis, suggesting these as potential biomarkers and therapeutic targets in these cases [71].

The pathological relevance of NLRP3 is also highlighted by its role in viral infections, where it mediates both antiviral responses and inflammation [72]. Viruses such as influenza A virus (IAV), dengue virus (DENV), human chikungunya virus (CHIKV), and severe acute respiratory syndrome coronavirus-2 (SARS-CoV-2), among others, have been shown to activate the NLRP3 inflammasome [72,73]. While activation can assist in controlling viral replication, dysregulated NLRP3 activity contributes to severe pathological outcomes during viral infections [73]. This dual role highlights the need for precision in targeting NLRP3 to balance immune response and inflammation.

Given its central role in mediating inflammation across various conditions, the NLRP3 inflammasome represents an important therapeutic target [67]. Inhibiting aberrant NLRP3 activation offers the potential to alleviate inflammation-driven pathology in diseases ranging from viral infections to metabolic and autoimmune disorders, as well as febrile seizures in children.

Mefenamic Acid: The Only Inhibitor of NLRP3 Inflammasome

Mefenamic acid stands out as a unique NSAID, the only NSAID that can inhibit the NLRP3 inflammasome [22]. This property is distinct from its COX-mediated anti-inflammatory effects, offering an additional mechanism to modulate inflammation. By targeting the NLRP3 inflammasome, mefenamic acid reduces the overproduction of pro-inflammatory cytokines such as IL-1β, which are central to various inflammatory and immune responses [22]. The dual action of mefenamic acid, combining COX inhibition with NLRP3 inflammasome inhibition, holds significant therapeutic potential in pediatric populations, especially for conditions associated with aberrant inflammasome activation [4].

In febrile seizures, which are characterized by increased NLRP3 and IL-1β activity, mefenamic acid may reduce cytokine levels and alleviate symptoms. Furthermore, its potential antiviral benefits through the modulation of inflammasome activity provide an intriguing basis for its use in viral infections affecting children. Experts recognize mefenamic acid's novel therapeutic potential in addressing inflammatory conditions characterized by dysregulated NLRP3 inflammasome activity. This unique mechanism opens avenues for its use in pediatric inflammatory conditions, broadening the scope of its application in febrile illnesses.

Neuroprotective Effect of Mefenamic Acid in Febrile Seizures and FIRES

Febrile seizures are the most common type of acute seizure in children, affecting approximately 2-14% of children aged six months to five years globally [71]. Traditionally, rapid-acting antiepileptics and antipyretics have been utilized during subsequent febrile episodes to mitigate adverse effects associated with continuous antiepileptic drug use [74]. As the NLRP3 inflammasome plays a significant role in the pathophysiology of febrile seizures, the ability of mefenamic acid to inhibit this inflammasome suggests it may help reduce the levels of pro-inflammatory cytokines responsible for triggering febrile seizures [4]. Additionally, as a potent antipyretic agent, mefenamic acid helps manage the febrile conditions that often precipitate febrile seizures, adding to its therapeutic utility.

Emerging evidence highlights the neuroprotective potential of mefenamic acid in managing febrile seizures. In the first human study exploring its neuroprotective role in adults, mefenamic acid administered at a dosage of 500 mg twice daily for six months demonstrated safety and efficacy [5]. The study revealed its ability to improve cognitive impairment and reverse memory loss and brain inflammation, specifically through its anti-inflammatory activity targeting the NLRP3 inflammasome pathway [5]. This evidence is particularly relevant for conditions like FIRES, where aberrant neuroinflammation contributes significantly to disease pathology. By attenuating the levels of IL-1β, a key mediator in neuroinflammation, mefenamic acid offers promise in reducing the severity and frequency of febrile seizures.

The dual anti-inflammatory, antipyretic, and neuroprotective effects of mefenamic acid not only position it as an effective treatment for febrile seizures but also broaden its potential application in managing other pediatric febrile illnesses characterized by heightened neuroinflammatory responses. Further research is warranted to solidify its role and optimize its therapeutic use in these conditions.

Evidence Gaps in Contraindications for NSAIDs in Viral Fevers

NSAIDs are widely used as analgesics and antipyretics in viral infections, where supportive care is the mainstay. Their impact on viral disease progression remains unclear, with studies reporting both antiviral and immune-modulatory benefits, as well as potential risks of unresolved symptoms or complications [75]. NSAIDs modulate immunity by stimulating T lymphocytes, nitric oxide (NO), and interferons while suppressing neutrophils, macrophages, and antibody production. In cytokine storm conditions, NSAIDs have been evaluated as adjuncts, but their diverse immune effects yield mixed outcomes [75].

A systematic review by von Philipsborn et al. analyzed 28 studies in adults and 42 studies in children, finding no severe adverse events associated with NSAID use in viral infections. However, the absence of evidence for harm does not equate to proof of safety, underscoring the need for further research [76]. Another systematic review and meta-analysis by Purssell and While, involving studies on malaria, viral respiratory infections, and varicella, reported that antipyretics did not slow fever resolution in children. On the contrary, antipyretic use was associated with significantly faster fever clearance, supporting their safety and efficacy in managing febrile episodes [77].

In the context of acute viral respiratory tract infections (ARTIs), NSAIDs have shown benefits in outpatient management, particularly for fever and sore throat. Although these drugs did not improve mortality or oxygenation outcomes in hospitalized patients, they were not linked to increased death rates or mechanical ventilation needs [78]. Also, during the COVID-19 pandemic, extensive reviews of clinical trials and observational studies found no evidence to justify restrictions on NSAID use. Instead, early NSAID intervention was associated with better symptom control and reduced complications, highlighting their potential to address excessive inflammatory responses and minimize inappropriate antibiotic use [79].

In summary, the contraindications against NSAID use in viral fevers lack robust evidence, and several studies suggest that NSAIDs can safely and effectively manage symptoms such as fever and inflammation. Also, NSAID contraindications are mainly documented in the context of aspirin. Emerging research points to their potential benefits in mitigating inflammatory responses in viral illnesses, particularly when used early. Expert opinion emphasizes the importance of reevaluating current guidelines and the need for high-quality studies that could enhance therapeutic strategies while avoiding unnecessary antibiotic prescriptions.

Promising Therapeutic Role of Mefenamic Acid in Viral Infections

Mefenamic acid can be used along with standard antiviral drugs for the treatment of viral fevers, with its antipyretic activity and supportive antiviral activity [6]. Its unique pharmacological properties, including antiviral and anti-inflammatory effects, make it a promising candidate for addressing viral pathologies [4]. Emerging research is highlighting the potential of mefenamic acid in managing viral infections such as dengue [80], chikungunya [81], and severe acute respiratory syndrome (SARS) [7], either alone or in combination with established antiviral agents. One proposed mechanism of action involves the inhibition of viral serine proteases. By targeting these proteases, mefenamic acid may block viral entry into host cells or prevent replication by interfering with the cleavage of viral polyproteins, which are essential for viral assembly and proliferation [6]. Additionally, the NLRP3 inflammasome inhibitory activity of mefenamic acid plays a crucial role in managing viral infections [72,73]. Although mefenamic acid demonstrates significant activity in inhibiting viral replication, its primary clinical application remains limited to its antipyretic and anti-inflammatory properties in managing infectious conditions. Further research is necessary to confirm its efficacy and optimize its application in clinical practice for the treatment of dengue in pediatric settings.

Mefenamic acid in dengue infection: Mefenamic acid is emerging as a potential therapeutic option for dengue, particularly for febrile illnesses. Studies suggest it possesses antiviral properties against the dengue virus, expanding its clinical applications beyond its traditional anti-inflammatory and antipyretic roles. Rothan et al. evaluated its antiviral activity against the dengue NS2B-NS3 protease (DENV2 NS2B-NS3pro), a key enzyme in viral replication [80]. The study demonstrated that mefenamic acid significantly inhibited dengue viral replication, effectively reducing viral RNA levels while maintaining a better safety profile than doxycycline [80].

Apart from the antiviral property, mefenamic acid also showed promise in dengue treatment by targeting the NLRP3 inflammasome, a key driver of inflammation and vascular leakage in severe dengue cases like dengue hemorrhagic fever (DHF) and dengue shock syndrome (DSS) [73,82,83]. Dengue virus proteins M, NS2A, and NS2B activate NLRP3, with NS2A and NS2B acting as viroporins that disrupt mitochondrial function, induce calcium imbalance, and promote reactive oxygen species (ROS) production, which are major triggers of NLRP3 activation [73,84,85]. This activation contributes to vascular leakage, thrombocytopenia, and platelet dysfunction via caspase-1-mediated IL-1β secretion and receptor-interacting protein 1/receptor-interacting protein 3 (RIP1/RIP3)-induced mitochondrial ROS [82,86,87]. Pharmacological inhibition of NLRP3, as seen in preclinical studies, alleviates inflammatory response, reduces platelet defects, and manages the severity of dengue infection [82,86].

In this context, the proposed antiviral and NLRP3 inflammasome inhibitory activity of mefenamic acid suggests that it may help alleviate clinical symptoms and support recovery in dengue infections. The current literature suggests that mefenamic acid could play a critical role in managing dengue infection, particularly in reducing the severity of complications like DHF and DSS, and highlights the need for further research to confirm its therapeutic potential.

Mefenamic acid in CHIKV infection: CHIKV infection remains a major global health concern due to the widespread distribution of its mosquito vectors and the lack of effective anti-CHIKV drugs [88]. Emerging evidence suggests that mefenamic acid, an NSAID, may play a crucial role in combating CHIKV. Rothan et al. demonstrated that mefenamic acid inhibits CHIKV replication by binding to viral proteases, essential enzymes in the viral life cycle [80]. Combining mefenamic acid with ribavirin significantly reduced CHIKV infection and its associated pathological signs in vivo, showcasing strong antiviral activity and potential for broader clinical applications. However, further clinical investigations are needed to validate these findings and optimize treatment protocols for CHIKV and other viral infections [81].

Repurposing of mefenamic acid in COVID-19: The COVID-19 pandemic led to the repurposing of NSAIDs, with mefenamic acid emerging as a promising candidate due to its ability to inhibit the NLRP3 inflammasome, a key driver of severe inflammation [7]. One of the most critical complications of COVID-19 is the cytokine storm, which results from uncontrolled pro-inflammatory cytokine production, leading to tissue damage and organ failure [89]. NLRP3 inflammasome activation, triggered by lysosomal disruption and ion imbalance, plays a major role in this process and offers a potential therapeutic target for mitigating the excessive immune response characteristic of severe COVID-19 [90,91].

Mefenamic acid specifically targets and inhibits the NLRP3 inflammasome, reducing the release of pro-inflammatory biomarkers, including C-reactive proteins (CRPs), and potentially preventing the onset of cytokine storms [92]. By effectively targeting key pathways, it disrupts the feedback loops responsible for the excessive cytokine release [79]. Mefenamic acid has also demonstrated antiviral activity by potentially inhibiting serine proteases, which may block viral entry or replication by interfering with the cleavage of viral polyproteins [6]. These properties support its use as an adjunctive treatment to manage symptoms and reduce severe immune complications, especially in early infection [4,93]. Clinical evidence also supports its adjunctive role in COVID-19 treatment. A phase II trial by Guzman-Esquivel et al. showed that mefenamic acid significantly reduced the time to reach post-acute sequelae of SARS-CoV-2 (PASS) and shortened symptom duration, including headache and sore throat [94]. Case reports have demonstrated its efficacy in reducing fever, managing thermo-dysregulation, and alleviating cytokine storms in mild to moderate cases [92]. Mefenamic acid has been safely used from the first day of infection to manage myalgia and reduce inflammation, particularly in patients with elevated CRP levels [7].

The experience of repurposing mefenamic acid in COVID-19 sets a case study and provides a valuable foundation for its application in future outbreaks of similar viral infections. Its role in reducing inflammation and viral replication, coupled with its established safety profile, highlights its potential as an essential tool in pandemic preparedness and response.

Possible Role of Mefenamic Acid in Children With Asthma

Mefenamic acid could play a dual role in managing asthma in children through its antagonistic effects on bradykinin and inhibition of the NLRP3 inflammasome. Bradykinin, a potent bronchoconstrictor, exacerbates asthma symptoms by promoting inflammation and the release of other inflammatory mediators like prostaglandins, which amplify airway sensitivity and inflammation [95,96]. Mefenamic acid could mitigate these effects by inhibiting prostaglandin production, thereby reducing tissue sensitivity to bradykinin and alleviating bronchoconstriction [97]. In contrast to ibuprofen, it does not cause bronchospasm.

In asthma, the NLRP3 inflammasome plays a role in airway inflammation by promoting the release of inflammatory cytokines such as IL-1β. Researchers have proposed that targeting the NLRP3 inflammasome may provide new treatment options for asthma management, specifically allergic asthma [98]. The ability of mefenamic acid to inhibit the NLRP3 inflammasome offers additional therapeutic potential by decreasing the airway inflammation mediated by IL-1β, a key cytokine involved in severe asthma.

Mefenamic Acid Beyond Fever, Inflammation, and Pain: Role in Cancer Therapy

Beyond its antipyretic, analgesic, and anti-inflammatory activity, mefenamic acid has also gained attention for its potential role in cancer therapy. Studies highlight its ability to induce apoptosis in cancer cells, particularly through the caspase-3 pathway in liver cancer cell lines [99]. Mefenamic acid enhances the efficacy of radiation therapy by amplifying the production of ROS, which sensitizes cancer cells, leading to increased apoptosis and reduced tumor growth [100]. Preclinical and in vivo studies indicate its efficacy in inhibiting tumor growth [101], particularly through targeting platelet-derived growth factor (PDGF) pathways and reducing angiogenesis in hepatocellular carcinoma [102]. Mefenamic acid has been shown to enhance cancer cell sensitivity to anticancer drugs by inhibiting aldo-keto reductase 1C, addressing drug resistance [103]. A trial in castration-resistant prostate cancer reported significant reductions in prostate-specific antigen (PSA) levels and improved patient outcomes, supporting its role as a potential adjunctive therapy [66]. These findings suggest that mefenamic acid warrants further investigation as a synergistic agent in oncology.

Consensus statements

The consensus statements presented in Table 4 summarize the collective expert opinion on the pharmacological properties, dosing guidelines, clinical applications, emerging evidence, and safety considerations of mefenamic acid. Each statement is presented with its respective level of agreement, highlighting the robustness and clinical relevance of these best-practice consensus statements.

**Table 4:** Consensus statements and level of agreement *Level of the agreement includes strongly agree and agree votes. NSAID: nonsteroidal anti-inflammatory drug; COX-2: cyclooxygenase-2; EP: E-type prostanoid; NLRP3: nucleotide-binding domain, leucine-rich-containing family, pyrin domain-containing-3; IL-1β: interleukin-1 beta; FIRES: febrile infection-related epilepsy syndrome

No.	Best-practice consensus statements	Level of agreement*
Pharmacological properties and mechanism of action		
1	Mefenamic acid, a member of the NSAID class, exhibits a unique mechanism of action, including preferential COX-2 inhibition, EP receptor blockade, and NLRP3 inflammasome inhibition, contributing to its comprehensive therapeutic effects.	100%
Dosing and administration		
2	Pediatric dosing of mefenamic acid is weight-based at 5 mg/kg body weight every 8 hours, with a suspension preferred for children under 12 years.	95%
3	Individualized, weight-based dosing of mefenamic acid minimizes the risks of under- or overdosing, ensuring safe and effective pediatric therapy.	95%
Clinical applications in fever and pain management		
4	Evidence supports using mefenamic acid in pediatric febrile illness, ensuring effective and sustained symptom relief.	100%
5	Clinical evidence demonstrates that mefenamic acid provides a faster onset and more sustained antipyretic effect compared to paracetamol and ibuprofen.	90%
6	Sustained antipyretic action for 8 hours reduces dosing frequency, improving compliance in pediatric care and promoting stable fever control in children.	85%
7	Broad-spectrum antipyretic, analgesic, and anti-inflammatory properties of mefenamic acid make it suitable for managing pain of various etiologies in pediatric patients.	85%
8	Mefenamic acid is the drug of choice in adolescent girls for dysmenorrhea and menorrhagia.	100%
9	In children with asthma, mefenamic acid is preferred over ibuprofen in high fever and pain management.	85%
Emerging evidence and potential indications		
10	The inhibition of the NLRP3 inflammasome and reduction of IL-1β levels by mefenamic acid represent a novel therapeutic option for managing febrile seizures and FIRES in children.	85%
11	Mefenamic acid has demonstrated antiviral properties in in vitro studies by inhibiting viral protease activity and reducing replication of dengue and chikungunya viruses, highlighting its potential for further exploring this role in clinical practice.	100%
12	Mefenamic acid may be used as antipyretic therapy in dengue, with minimal risk of bleeding complications compared to ibuprofen due to its effect on platelet function.	75%
Safety considerations		
13	The preferential inhibition of COX-2 by mefenamic acid minimizes gastrointestinal side effects compared to non-selective NSAIDs, enhancing its safety profile in pediatric use.	85%
14	The administration of mefenamic acid with food and adequate hydration reduces the risk of adverse gastrointestinal events in children.	100%

## Conclusions

This consensus paper provides a foundational framework to support the safe and effective integration of mefenamic acid into pediatric practice, encouraging informed clinical decision-making and improved patient outcomes. These consensus highlights the critical role of mefenamic acid in managing fever and pain in pediatric practice, emphasizing its unique pharmacological actions, sustained antipyretic effects, and broad clinical applications. Its preferential COX-2 inhibition, EP receptor blockade, and NLRP3 inflammasome inhibition offer significant therapeutic advantages, making it an effective and versatile option in pediatric care. Moreover, emerging evidence highlights its potential in managing febrile seizures and viral infections, further expanding its utility. Despite these strengths, challenges persist, including limited large-scale pediatric studies and misconceptions regarding safety. Addressing these gaps through robust clinical trials and comprehensive guidelines will enable a more consistent and evidence-based approach to its use. Future research should aim for adequately powered studies, with minimum sample sizes to ensure statistically robust conclusions regarding safety and efficacy. Focused research is particularly needed in children aged six months to five years, a group for whom clinical evidence on the use of mefenamic acid is limited. The long-term safety of mefenamic acid in children is still unclear, due to the limited availability of robust, longitudinal safety data. Standardization of outcome measures, including the use of pediatric-validated pain scales, organ-specific safety assessments, and long-term follow-up protocols, is essential to enable meaningful comparisons across studies. A coordinated research framework is recommended, with short-term objectives (within 2-3 years) focused on accumulating safety data and medium-term goals (3-5 years) directed toward evaluating efficacy and comparative effectiveness to address current knowledge gaps.

## Appendices

Literature search strategy

Databases searched: MEDLINE, Embase, and Google Scholar

Search time frame: January 2011 to November 2024

Language limit: English only

Key search terms used (individually and in combinations)

o   "Mefenamic acid"

o   "Mefenamic acid AND pediatric"

o   "Mefenamic acid AND fever"

o   "Mefenamic acid AND children"

o   "NSAIDs AND pediatric fever"

o   "Mefenamic acid AND pharmacokinetics"

o   "Mefenamic acid AND safety"

o   "Mefenamic acid AND febrile seizures"

o   "Mefenamic acid AND dengue"

o   "Mefenamic acid AND chikungunya"

o   "Mefenamic acid AND COVID-19"

o   "NLRP3 inflammasome AND mefenamic acid"

o   "NSAIDs AND platelet function"

o   "NSAIDs AND renal safety in children"

Additional methods

o   Backward citation tracking of key articles

o   Manual screening of references from major review articles

## References

[1] de Bont EG, Lepot JM, Hendrix DA, Loonen N, Guldemond-Hecker Y, Dinant GJ, Cals JW (2015). Workload and management of childhood fever at general practice out-of-hours care: an observational cohort study. BMJ Open.

[2] Gambhir A, Tamboli S, Prasad SM, Inamdar NR (2021). Pyrogenic cytokines mediated pathophysiology of fever and role of mefenamic acid in pediatric practice. Indian J Clin Pract.

[3] Bertille N, Fournier-Charrière E, Pons G, Chalumeau M (2013). Managing fever in children: a national survey of parents' knowledge and practices in France. PLoS One.

[4] Tripathi SK, Kasture PN (2021). Mefenamic acid: the evolution of a versatile NSAID. Indian J Clin Pract.

[5] Melnikov V, Tiburcio-Jimenez D, Mendoza-Hernandez MA (2021). Improve cognitive impairment using mefenamic acid non-steroidal anti-inflammatory therapy: additional beneficial effect found in a controlled clinical trial for prostate cancer therapy. Am J Transl Res.

[6] Pareek RP (2020). Use of mefenamic acid as a supportive treatment of COVID-19: a repurposing drug. Int J Sci Res.

[7] Aggarwal KK, Woei Chong Y, Sharma R (2021). Repurposing mefenamic acid in the management of COVID-19. J Indian Med Assoc.

[8] Gottlieb M, Caretta-Weyer H, Chan TM, Humphrey-Murto S (2023). Educator's blueprint: a primer on consensus methods in medical education research. AEM Educ Train.

[9] Nasa P, Jain R, Juneja D (2021). Delphi methodology in healthcare research: how to decide its appropriateness. World J Methodol.

[10] Cimolai N (2013). The potential and promise of mefenamic acid. Expert Rev Clin Pharmacol.

[11] Collier HO, Sweatman WJ (1968). Antagonism by fenamates of prostaglandin F2α and of slow reacting substance on human bronchial muscle. Nature.

[12] Levy B, Lindner HR (1971). Selective blockade of the vasodepressor response to prostaglandin F2α in the anaesthetized rabbit. Br J Pharmacol.

[13] Burka JF, Eyre P (1974). Studies of prostaglandins and prostaglandin antagonists on bovine pulmonary vein in vitro. Prostaglandins.

[14] Cryer B, Feldman M (1998). Cyclooxygenase-1 and cyclooxygenase-2 selectivity of widely used nonsteroidal anti-inflammatory drugs. Am J Med.

[15] Blomqvist A, Engblom D (2018). Neural mechanisms of inflammation-induced fever. Neuroscientist.

[16] Dantzer R, Konsman JP, Bluthé RM, Kelley KW (2000). Neural and humoral pathways of communication from the immune system to the brain: parallel or convergent?. Auton Neurosci.

[17] Ricciotti E, FitzGerald GA (2011). Prostaglandins and inflammation. Arterioscler Thromb Vasc Biol.

[18] Chavan SS, Pavlov VA, Tracey KJ (2017). Mechanisms and therapeutic relevance of neuro-immune communication. Immunity.

[19] López Bernal A, Buckley S, Rees CM, Marshall JM (1991). Meclofenamate inhibits prostaglandin E binding and adenylyl cyclase activation in human myometrium. J Endocrinol.

[20] Daniels MJ, Rivers-Auty J, Schilling T (2016). Fenamate NSAIDs inhibit the NLRP3 inflammasome and protect against Alzheimer's disease in rodent models. Nat Commun.

[21] Chen GL, Zeng B, Eastmond S, Elsenussi SE, Boa AN, Xu SZ (2012). Pharmacological comparison of novel synthetic fenamate analogues with econazole and 2-APB on the inhibition of TRPM2 channels. Br J Pharmacol.

[22] Kasture P, Mehta K, Gowda A (2022). Inflammasome, inflammation, infection and mefenamic acid. J Assoc Physicians India.

[23] Kunkulol RR, Chavan AU, Chavva AK (2013). Evaluation of efficacy and tolerability of acetaminophen (paracetamol) and mefenamic acid as antipyretic in pediatric patients with febrile illness: a comparative study. Int J Med Res Health Sci.

[24] McGettigan P, Henry D (2000). Current problems with non-specific COX inhibitors. Curr Pharm Des.

[25] Williams BS, Buvanendran A (2011). Nonopioid analgesics: NSAIDs, COX-2 inhibitors, and acetaminophen. Essentials of Pain Medicine.

[26] Farkouh A, Hemetsberger M, Noe CR, Baumgärtel C (2022). Interpreting the benefit and risk data in between-drug comparisons: illustration of the challenges using the example of mefenamic acid versus ibuprofen. Pharmaceutics.

[27] Langford RA, Hogg M, Bjorksten AR, Williams DL, Leslie K, Jamsen K, Kirkpatrick C (2016). Comparative plasma and cerebrospinal fluid pharmacokinetics of paracetamol after intravenous and oral administration. Anesth Analg.

[28] Ayoub SS (2021). Paracetamol (acetaminophen): a familiar drug with an unexplained mechanism of action. Temperature (Austin).

[29] Kulo A, Peeters MY, Allegaert K (2013). Pharmacokinetics of paracetamol and its metabolites in women at delivery and post-partum. Br J Clin Pharmacol.

[30] Athersuch TJ, Antoine DJ, Boobis AR (2018). Paracetamol metabolism, hepatotoxicity, biomarkers and therapeutic interventions: a perspective. Toxicol Res (Camb).

[31] (2014). Martindale: The Complete Drug Reference. Pharmaceutical Press: London, UK.

[32] Rainsford KD (2009). Ibuprofen: pharmacology, efficacy and safety. Inflammopharmacology.

[33] Green R, Webb D, Jeena PM (2021). Management of acute fever in children: consensus recommendations for community and primary healthcare providers in sub-Saharan Africa. Afr J Emerg Med.

[34] (2023). MEFTAL®-P suspension. https://meftal.com/assets/MEFTAL-P-Suspension.pdf.

[35] (2022). MEFTAL®-250 DT and MEFTAL®-500 tablets. https://meftal.com/assets/MEFTAL-250-DT.pdf.

[36] Moore RA, Derry S, Wiffen PJ, Straube S (2015). Effects of food on pharmacokinetics of immediate release oral formulations of aspirin, dipyrone, paracetamol and NSAIDs - a systematic review. Br J Clin Pharmacol.

[37] (2023). Mefenamic acid 50 mg/5 ml suspension. https://www.medicines.org.uk/emc/product/13316/smpc.

[38] Sullivan JE, Farrar HC (2011). Fever and antipyretic use in children. Pediatrics.

[39] Milani GP, Alberti I, Bonetti A, Garattini S, Corsello A, Marchisio P, Chiappini E (2024). Definition and assessment of fever-related discomfort in pediatric literature: a systematic review. Eur J Pediatr.

[40] Reddy GT, Gobbur RH, Patil SV (2020). Randomized open-label study to compare the safety and efficacy of paracetamol, ibuprofen, and mefenamic acid in febrile children. Int J Sci Stud.

[41] Khubchandani RP, Ghatikar KN, Keny S, Usgaonkar NG (1995). Choice of antipyretic in children. J Assoc Physicians India.

[42] Loya A, Siddiqui MS, Sangle A, Ingale V, Saha S, Nelanuthala M (2022). The antipyretic effect of high-dose paracetamol versus mefenamic acid in the treatment of febrile children: a randomized control trial. Cureus.

[43] Dabholkar KM (2002). Mefenamic acid is an effective and well tolerated antipyretic for children. Ind Pract.

[44] Pasi R, Babu TA, Kallidoss VK (2023). Efficacy of oral mefenamic acid versus paracetamol as a prophylactic analgesic for needle pain in children receiving vaccination: a three-arm, parallel, triple-blind, placebo-controlled MAP VaC randomized controlled trial. Ther Adv Vaccines Immunother.

[45] Prymula R, Siegrist CA, Chlibek R (2009). Effect of prophylactic paracetamol administration at time of vaccination on febrile reactions and antibody responses in children: two open-label, randomised controlled trials. Lancet.

[46] Feng X, Wang X (2018). Comparison of the efficacy and safety of non-steroidal anti-inflammatory drugs for patients with primary dysmenorrhea: a network meta-analysis. Mol Pain.

[47] Hamaguchi T, Shinkuma D, Yamanaka Y, Mizuno N (1986). Bioavailability of mefenamic acid: influence of food and water intake. J Pharm Sci.

[48] Lai EC, Shin JY, Kubota K (2018). Comparative safety of NSAIDs for gastrointestinal events in Asia-Pacific populations: a multi-database, international cohort study. Pharmacoepidemiol Drug Saf.

[49] Lucas GN, Leitão AC, Alencar RL, Xavier RM, Daher EF, Silva Junior GB (2019). Pathophysiological aspects of nephropathy caused by non-steroidal anti-inflammatory drugs. J Bras Nefrol.

[50] Hörl WH (2010). Nonsteroidal anti-inflammatory drugs and the kidney. Pharmaceuticals (Basel).

[51] Kamour A, Crichton S, Cooper G (2017). Central nervous system toxicity of mefenamic acid overdose compared with other NSAIDs: an analysis of cases reported to the United Kingdom National Poisons Information Service. Br J Clin Pharmacol.

[52] Khansari PS, Halliwell RF (2019). Mechanisms underlying neuroprotection by the NSAID mefenamic acid in an experimental model of stroke. Front Neurosci.

[53] Salmanzadeh H, Halliwell RF (2024). Antiseizure properties of fenamate NSAIDs determined in mature human stem-cell derived neuroglial circuits. Front Pharmacol.

[54] Halliwell RF, Thomas P, Patten D, James CH, Martinez-Torres A, Miledi R, Smart TG (1999). Subunit-selective modulation of GABAA receptors by the non-steroidal anti-inflammatory agent, mefenamic acid. Eur J Neurosci.

[55] Khansari PS, Halliwell RF (2009). Evidence for neuroprotection by the fenamate NSAID, mefenamic acid. Neurochem Int.

[56] Song L, Pei L, Yao S, Wu Y, Shang Y (2017). NLRP3 inflammasome in neurological diseases, from functions to therapies. Front Cell Neurosci.

[57] Dillon P, O'Brien KK, McDonnell R (2015). Prevalence of prescribing in pregnancy using the Irish primary care research network: a pilot study. BMC Pregnancy Childbirth.

[58] (2001). The transfer of drugs and other chemicals into human milk. Pediatrics.

[59] Buchanan RA, Eaton CJ, Koeff ST, Kinkel AW (1968). The breast milk excretion of mefenamic acid. Curr Ther Res Clin Exp.

[60] Bloor M, Paech M (2013). Nonsteroidal anti-inflammatory drugs during pregnancy and the initiation of lactation. Anesth Analg.

[61] Davis A, Robson J (2016). The dangers of NSAIDs: look both ways. Br J Gen Pract.

[62] Schafer AI (1995). Effects of nonsteroidal antiinflammatory drugs on platelet function and systemic hemostasis. J Clin Pharmacol.

[63] Chelucci RC, Dutra LA, Lopes Pires ME, de Melo TR, Bosquesi PL, Chung MC, Dos Santos JL (2014). Antiplatelet and antithrombotic activities of non-steroidal anti-inflammatory drugs containing an N-acyl hydrazone subunit. Molecules.

[64] Knijff-Dutmer EA, Kalsbeek-Batenburg EM, Koerts J, van de Laar MA (2002). Platelet function is inhibited by non-selective non-steroidal anti-inflammatory drugs but not by cyclo-oxygenase-2-selective inhibitors in patients with rheumatoid arthritis. Rheumatology (Oxford).

[65] van Eijkeren MA, Christiaens GC, Geuze HJ, Haspels AA, Sixma JJ (1992). Effects of mefenamic acid on menstrual hemostasis in essential menorrhagia. Am J Obstet Gynecol.

[66] Guzman-Esquivel J, Mendoza-Hernandez MA, Tiburcio-Jimenez D (2020). Decreased biochemical progression in patients with castration-resistant prostate cancer using a novel mefenamic acid anti-inflammatory therapy: a randomized controlled trial. Oncol Lett.

[67] Wang Z, Zhang S, Xiao Y (2020). NLRP3 inflammasome and inflammatory diseases. Oxid Med Cell Longev.

[68] Zhang X, Wang Z, Zheng Y (2023). Inhibitors of the NLRP3 inflammasome pathway as promising therapeutic candidates for inflammatory diseases (review). Int J Mol Med.

[69] Awad F, Assrawi E, Louvrier C (2018). Inflammasome biology, molecular pathology and therapeutic implications. Pharmacol Ther.

[70] Kwon A, Kwak BO, Kim K (2018). Cytokine levels in febrile seizure patients: a systematic review and meta-analysis. Seizure.

[71] Liu Z, Xian H, Ye X, Chen J, Ma Y, Huang W (2020). Increased levels of NLRP3 in children with febrile seizures. Brain Dev.

[72] Chen IY, Ichinohe T (2015). Response of host inflammasomes to viral infection. Trends Microbiol.

[73] Shrivastava G, Valenzuela Leon PC, Calvo E (2020). Inflammasome fuels dengue severity. Front Cell Infect Microbiol.

[74] Offringa M, Newton R, Nevitt SJ, Vraka K (2021). Prophylactic drug management for febrile seizures in children. Cochrane Database Syst Rev.

[75] Gerges RH (2022). NSAIDs: a double edged sword in viral infections. Int J Med Rev.

[76] von Philipsborn P, Biallas R, Burns J (2020). Adverse effects of non-steroidal anti-inflammatory drugs in patients with viral respiratory infections: rapid systematic review. BMJ Open.

[77] Purssell E, While AE (2013). Does the use of antipyretics in children who have acute infections prolong febrile illness? A systematic review and meta-analysis. J Pediatr.

[78] Azh N, Barzkar F, Motamed-Gorji N (2022). Nonsteroidal anti-inflammatory drugs in acute viral respiratory tract infections: an updated systematic review. Pharmacol Res Perspect.

[79] Bassetti M, Andreoni M, Santus P, Scaglione F (2024). NSAIDs for early management of acute respiratory infections. Curr Opin Infect Dis.

[80] Rothan HA, Buckle MJ, Ammar YA, Mohammadjavad P, Shatrah O, Noorsaadah AR, Rohana Y (2013). Study the antiviral activity of some derivatives of tetracycline and non-steroid anti inflammatory drugs towards dengue virus. Trop Biomed.

[81] Rothan HA, Bahrani H, Abdulrahman AY (2016). Mefenamic acid in combination with ribavirin shows significant effects in reducing chikungunya virus infection in vitro and in vivo. Antiviral Res.

[82] Pan P, Zhang Q, Liu W (2019). Dengue virus M protein promotes NLRP3 inflammasome activation to induce vascular leakage in mice. J Virol.

[83] Jaiyen Y, Masrinoul P, Kalayanarooj S, Pulmanausahakul R, Ubol S (2009). Characteristics of dengue virus-infected peripheral blood mononuclear cell death that correlates with the severity of illness. Microbiol Immunol.

[84] Shrivastava G, García-Cordero J, León-Juárez M, Oza G, Tapia-Ramírez J, Villegas-Sepulveda N, Cedillo-Barrón L (2017). NS2A comprises a putative viroporin of dengue virus 2. Virulence.

[85] Shrivastava G, Visoso-Carvajal G, Garcia-Cordero J (2020). Dengue virus serotype 2 and its non-structural proteins 2A and 2B activate NLRP3 inflammasome. Front Immunol.

[86] Lien TS, Chan H, Sun DS, Wu JC, Lin YY, Lin GL, Chang HH (2021). Exposure of platelets to dengue virus and envelope protein domain III induces NLRP3 inflammasome-dependent platelet cell death and thrombocytopenia in mice. Front Immunol.

[87] Hottz ED, Lopes JF, Freitas C (2013). Platelets mediate increased endothelium permeability in dengue through NLRP3-inflammasome activation. Blood.

[88] Silva LA, Dermody TS (2017). Chikungunya virus: epidemiology, replication, disease mechanisms, and prospective intervention strategies. J Clin Invest.

[89] Jarczak D, Nierhaus A (2022). Cytokine storm-definition, causes, and implications. Int J Mol Sci.

[90] Shah A (2020). Novel coronavirus-induced NLRP3 inflammasome activation: a potential drug target in the treatment of COVID-19. Front Immunol.

[91] Zhao N, Di B, Xu LL (2021). The NLRP3 inflammasome and COVID-19: activation, pathogenesis and therapeutic strategies. Cytokine Growth Factor Rev.

[92] Aggarwal KK (2021). Mefenamic acid as steroid-sparing antiinflammatory drug during viral phase of COVID-19: 5 case reports. Indian J Clin Pract.

[93] Nainu F, Mamada SS, Emran TB (2023). Prospective role of NSAIDs with antiviral properties for pharmacological management of postsurgical procedures during COVID-19. Int J Surg.

[94] Guzman-Esquivel J, Galvan-Salazar HR, Guzman-Solorzano HP (2022). Efficacy of the use of mefenamic acid combined with standard medical care vs. standard medical care alone for the treatment of COVID‑19: a randomized double‑blind placebo‑controlled trial. Int J Mol Med.

[95] Ricciardolo FL, Folkerts G, Folino A, Mognetti B (2018). Bradykinin in asthma: modulation of airway inflammation and remodelling. Eur J Pharmacol.

[96] Kharitonov SA, Sapienza MM, Chung KF, Barnes PJ (1999). Prostaglandins mediate bradykinin-induced reduction of exhaled nitric oxide in asthma. Eur Respir J.

[97] CO HO, SH PG (1963). Antagonism by mefenamic and flufenamic acids of the bronchoconstrictor action of kinins in the guinea-pig. Br J Pharmacol Chemother.

[98] Ma M, Li G, Qi M, Jiang W, Zhou R (2021). Inhibition of the inflammasome activity of NLRP3 attenuates HDM-induced allergic asthma. Front Immunol.

[99] Woo DH, Han IS, Jung G (2004). Mefenamic acid-induced apoptosis in human liver cancer cell-lines through caspase-3 pathway. Life Sci.

[100] Hosseinimehr SJ, Safavi Z, Kangarani Farahani S, Noaparst Z, Ghasemi A, Asgarian-Omran H (2019). The synergistic effect of mefenamic acid with ionizing radiation in colon cancer. J Bioenerg Biomembr.

[101] Seyyedi R, Talebpour Amiri F, Farzipour S, Mihandoust E, Hosseinimehr SJ (2022). Mefenamic acid as a promising therapeutic medicine against colon cancer in tumor-bearing mice. Med Oncol.

[102] Patel SS, Tripathi R, Chavda VK, Savjani JK (2020). Anticancer potential of mefenamic acid derivatives with platelet-derived growth factor inhibitory property. Anticancer Agents Med Chem.

[103] Shiiba M, Yamagami H, Yamamoto A (2017). Mefenamic acid enhances anticancer drug sensitivity via inhibition of aldo-keto reductase 1C enzyme activity. Oncol Rep.

